# *Anaplasma phagocytophilum* prevalence in ticks and rodents in an urban and natural habitat in South-Western Slovakia

**DOI:** 10.1186/s13071-015-0880-8

**Published:** 2015-05-17

**Authors:** Zuzana Svitálková, Danka Haruštiaková, Lenka Mahríková, Lenka Berthová, Mirko Slovák, Elena Kocianová, Mária Kazimírová

**Affiliations:** Institute of Zoology, Slovak Academy of Sciences, Dúbravská cesta 9, 845 06 Bratislava, Slovakia; Institute of Biostatistics and Analyses, Faculty of Medicine and Faculty of Science, Masaryk University, Kamenice 3, 625 00 Brno, Czech Republic; Institute of Virology, Slovak Academy of Sciences, Dúbravská cesta 9, 845 05 Bratislava, Slovakia

**Keywords:** *Anaplasma phagocytophilum*, *Ixodes ricinus*, Rodents, Tick-borne pathogen

## Abstract

**Background:**

*Ixodes ricinus* is the principal vector of *Anaplasma phagocytophilum,* the ethiological agent of granulocytic anaplasmosis in Europe. Anaplasmosis is an emerging zoonotic disease with a natural enzootic cycle. The reservoir competence of rodents is unclear. Monitoring of *A. phagocytophilum* prevalence in *I. ricinus* and rodents in various habitat types of Slovakia may contribute to the knowledge about the epidemiology of anaplasmosis in Central Europe.

**Methods:**

Over 4400 questing ixodid ticks, 1000 rodent-attached ticks and tissue samples of 606 rodents were screened for *A. phagocytophilum* DNA by real-time PCR targeting the *msp2* gene. Ticks and rodents were captured along six transects in an urban/suburban and natural habitat in south-western Slovakia during 2011–2014. Estimates of wildlife (roe deer, red deer, fallow deer, mouflon, wild boar) densities in the study area were taken from hunter’s yearly reports. Spatial and temporal differences in *A. phagocytophilum* prevalence in questing *I. ricinus* and relationships with relative abundance of ticks and wildlife were analysed.

**Results:**

Overall prevalence of *A. phagocytophilum* in questing *I. ricinus* was significantly higher in the urban/suburban habitat (7.2 %; 95 % CI: 6.1–8.3 %) compared to the natural habitat (3.1 %; 95 % CI: 2.5–3.9 %) (*χ*^2^ = 37.451; *P* < 0.001). Significant local differences in prevalence of infected questing ticks were found among transects within each habitat as well as among years and between seasons. The trapped rodents belonged to six species. *Apodemus flavicollis* and *Myodes glareolus* prevailed in both habitats, *Microtus arvalis* was present only in the natural habitat. *I. ricinus* comprised 96.3 % of the rodent-attached ticks, the rest were *Haemaphysalis concinna*, *Ixodes trianguliceps* and *Dermacentor reticulatus*. Only 0.5 % of rodent skin and 0.6 % of rodent-attached ticks (only *I. ricinus*) were infected with *A. phagocytophilum*. Prevalence of *A. phagocytophilum* in questing *I. ricinus* did not correlate significantly with relative abundance of ticks or with abundance of wildlife in the area.

**Conclusion:**

The study confirms that urban *I. ricinus* populations are infected with *A. phagocytophilum* at a higher rate than in a natural habitat of south-western Slovakia and suggests that rodents are not the main reservoirs of the bacterium in the investigated area.

## Background

*Anaplasma phagocytophilum* is a medically and veterinary important emerging tick-borne pathogen in Europe. It is a gram-negative intracytoplasmic bacterium localized in the blood cells (primarily granulocytes) or endothelial cells of blood vessels [1] and causes febrile disease in humans (human granulocytic anaplasmosis—HGA) and animals (pasture fever, equine and canine granulocytic anaplasmosis) in areas of the northern hemisphere with endemic occurrence of *Ixodes* spp. ticks [1, 2]. The epidemiology of HGA and its risk to public health in Europe is still unclear as epidemiological data on the disease are scarce and most human infections probably result in minimal or no clinical manifestations [3, 4]. In Slovakia, only one clinical HGA case has been confirmed [5].

*Anaplasma phagocytophilum* has been detected in a broad range of vertebrate species including rodents, however, reservoir competence has been confirmed for a few species only [3, 6–15]. In Europe, *A. phagocytophilum* is primarily transmitted by *Ixodes ricinus,* the vector of a number of zoonotic microbial pathogens [16, 17]. Transovarial transmission in ixodid ticks has not been confirmed, therefore, the dependence on reservoir vertebrate hosts for maintenance of the infection in nature seems crucial.

Prevalence of *A. phagocytophilum* in ticks and vertebrate hosts and maintenance of transmission cycles depend on a wide range of abiotic and biotic factors of the environment [3, 12, 18]. Anthropogenic impact is very important as it results in changing landscape and land use patterns, affecting alterations in abundance of tick populations as well as changes in abundance and diversity of tick maintenance and reservoir vertebrate hosts [3, 4]. As a consequence, different *A. phagocytophilum* prevalences in questing ticks were found in urban, rural and natural habitats of Central European countries, with relatively high infection rates in green urban areas [4, 18–20].

In the USA, two *A. phagocytophilum* strains were reported. The Ap-ha variant can infect humans and its ecology is associated with rodents as reservoirs, whereas the Ap-1 variant circulates between *Ixodes scapularis* ticks and free-living ungulates, but not rodents [21, 22]. In contrast, the genetic variability and ecology of *A. phagocytophilum* in Europe is more complex [3] and rodents do not display high zoonotic potential [7, 14, 23]. In England, a distinct *A. phagocytophilum* strain not pathogenic for humans was found to be associated with rodents and transmitted by the endophilic *Ixodes trianguliceps* tick, whereas another non-rodent associated strain is transmitted by *I. ricinus* [24]. Recent studies supported the theory of two enzootic co-existing cycles for different *A. phagocytophilum* strains circulating in Central Europe [13, 25]. In general, a high degree of genetic diversity of *A. phagocytophilum* strains associated with *I. ricinus* and different vertebrate host species has been described [3, 13, 24, 26–29]. To assess the acarological risk of exposure of humans and domestic animals to potentially pathogenic strains, further investigations on prevalence and distribution of various *A. phagocytophilum* ecotypes in different habitat types of Europe are needed.

The objectives of the present study were: (1) Comparison of infection rates of *A. phagocytophilum* in questing *I. ricinus* ticks from habitats in south-western Slovakia that are differently affected by human intervention; (2) Assessment of the role of rodents in the transmission cycle of *A. phagocytophilum*; (3) Evaluation of the effect of wildlife density in the study area on prevalence of *A. phagocytophilum* in questing ticks*.*

## Methods

### Study area

**Figure 1:**
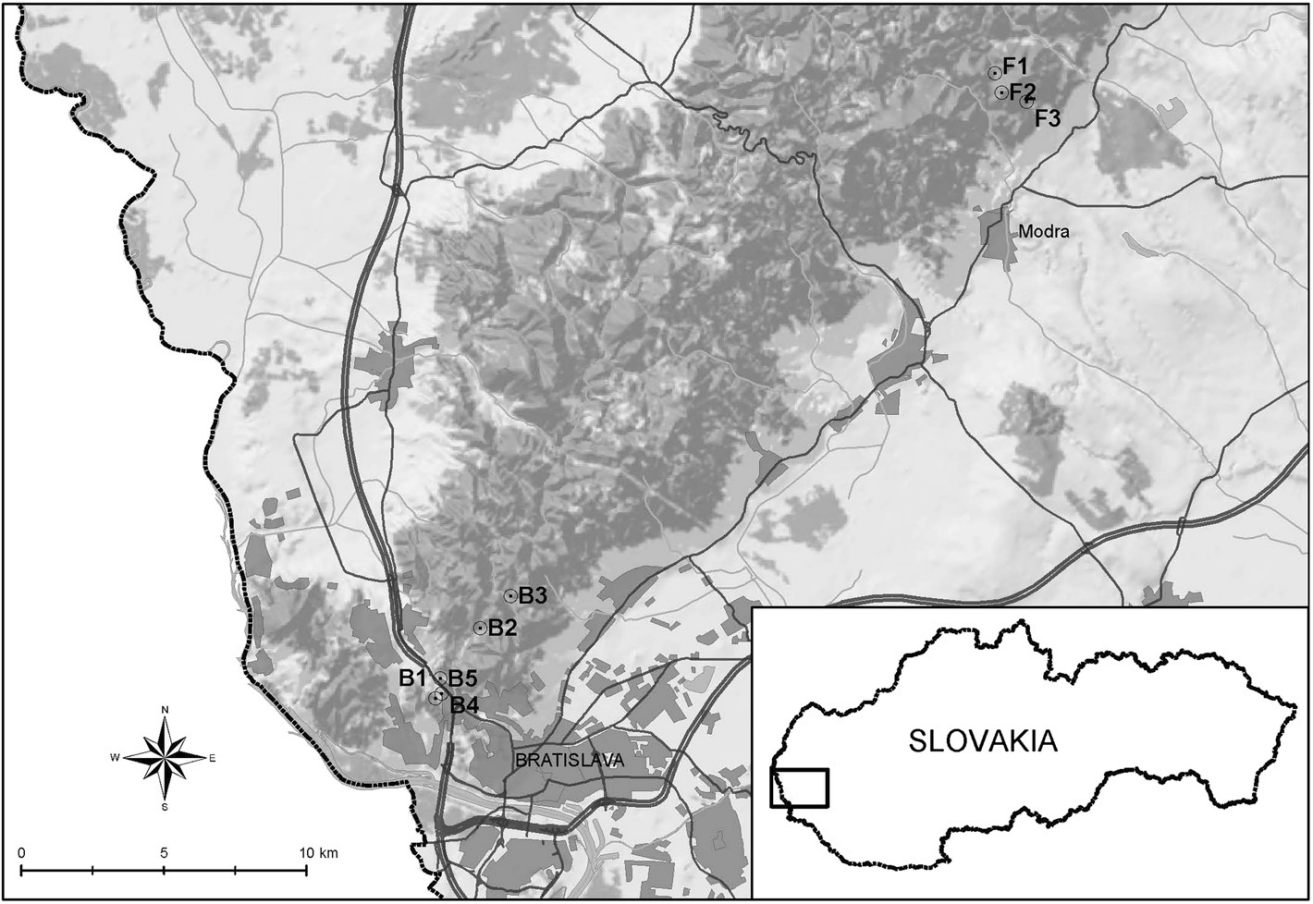
Map of the sampling locations in south-western Slovakia. Legend: B1-B5: transects in the urban/suburban site Bratislava; F1-F3: transects in the natural site Fúgelka

The study area is located in the Small Carpathian Mountains (south-western Slovakia) and comprises two different sites: Bratislava (48.17–48.20°N, 17.07–17.10°E, altitude 202–334 m a.s.l.) and Fúgelka (48.37–48.38°N, 17.30–17.32°E, altitude 336–436 m a.s.l.). In each site three 100 m long transects were selected for questing tick collections and rodent trapping (Fig. 1).

Site Bratislava is part of an urban/suburban area and includes the campus of the Slovak Academy of Sciences (SAS) and a recreational forest park (Železná studnička). It is located at the southern foothills of the Small Carpathian Mountains in the northern part of Bratislava town, i.e. in an area with significant anthropogenic impact and high frequency of visitors. The SAS campus (comprising transect B1) is an enclosed urban site with built-up plots. Transect B1 is located in a patch of forest close to the fence separating the SAS campus from the Bratislava Zoo. The main representatives of the fauna are small mammals, squirrels, hedgehogs, bats, feral cats, roe deer, lizards and birds. Red fox occasionally enter the location. The estimated roe deer density in the SAS campus is 30 ind./100 ha. Železná studnička (comprising transects B2 and B3) is part of Bratislava Forest Park located at the south-western foothills of the Small Carpathian Mountains and serves as a recreational and dog walking area. The overall airline distance of B2 and B3 from B1 is about 3 and 5 km, respectively. A variety of wildlife lives in the park. The estimated densities of hunted wildlife, based on annual censuses data in hunter’s yearly reports provided by the Ministry of Agriculture and Rural Development of the Slovak Republic, in the Forest Park are as follows (number of ind./100 ha): roe deer (*Capreolus capreolus*; 4.5), red deer (*Cervus elaphus*; 0.7), fallow deer (*Dama dama*; 0.7), mouflon (*Ovis musimon*; 0.9), wild boar (*Sus scrofa*; 1.7).

In addition to transects B1–B3, two transects (B4 and B5) were selected for random tick collections in Bratislava. B4 is a 150 m long transect located along the border of the SAS campus and a road, with ruderal vegetation, solitary deciduous trees and patches of mown grass. It is located 350 m apart from transect B1. Roe deer living in the SAS campus and their resting places were frequently encountered there. B5 is a 200 m long transect along fences of gardens and cottages outside the SAS campus. The fauna at this location is represented mainly by lizards, birds, small mammals and feral cats. The presence of wild ruminants or wild boar was not observed there. The airline distance of transect B5 from B4 is ca. 420 m and from transect B2 2.2 km (see Fig. 1).

Fúgelka is a natural site located in a non-fragmented deciduous forest, at a distance of about 40 km from Bratislava. Transects at Fúgelka are spaced approximately at the same distance (ca 2 km). Transect F1 is situated at the south-western foothills of the Small Carpathian Mountains, on the edge of a meadow and the forest, where wildlife are fed. Transects F2 and F3 are located in the forest along footpaths at the south-eastern foothills of the Small Carpathian Mountains. Fúgelka is affected by human activities to a lower degree than Bratislava, the frequency of visitors is relatively low, limited to tourists, rangers, foresters and hunters. The estimated densities of hunted wildlife (number of ind./100 ha) is as follows: roe deer (3.6), red deer (1.6), fallow deer (1.3), mouflon (4.2), wild boar (2.2).

### Questing ticks

Questing ticks were dragged with a 1 m^2^ sized blanket along the six transects in monthly intervals during the periods of highest activity of *I. ricinus*: April—June and September—October 2011–2013. The periods were chosen according to tick questing activity data which were obtained for SW Slovakia in the frame of the EDEN 6FP project during 2006–2008 (unpublished). At low tick densities in transects, random collections were carried out in the vicinity of transects to increase numbers of ticks for molecular screenings. In transects B4 and B5, random collections were carried out in April—June 2011. Collected ticks were stored in 70 % ethanol and identified to species level in the laboratory by using the identification key by Siuda [30]. Larvae were sampled, but only adults and nymphs of *I. ricinus* were included in the statistical analyses. Specimens of the other tick species collected were included in molecular screenings.

### Rodents and rodent-attached ticks

Rodents were live-trapped using Swedish bridge metal traps following the protocol of Stanko [31] in lines along the tick collection transects during spring (April—June) and autumn (September – October) of 2012, 2013 and 2014. The traps, baited with oat flakes, were placed 5 m apart, and each line was exposed during two consecutive nights, in total for 1800 trap nights in the urban/suburban habitat and 1900 trap nights in the natural habitat. Traps were checked regularly every morning. Captured rodents were transported to the laboratory, anaesthetized and sacrificed humanely. Rodent trapping and handling comply with current laws of the Slovak Republic and were approved by the Ministry of Environment of the Slovak Republic, Regional Environmental Office in Bratislava (licence ZPO-594/2012-SAB). Rodents were examined for ectoparasites, which were stored in 70 % ethanol and identified. At higher tick infestation rates, from each rodent individual at least five ticks per developmental stage and per species were selected for molecular screening. Rodent organs/tissues were dissected. Spleen was stored in 70 % ethanol and ear skin at −80 °C.

### DNA extraction

Macherey-Nagel NucleoSpin® Tissue kit (Düren, Germany) was used for DNA extraction. Rodent tissues (spleen, skin), questing and engorged ticks were disrupted by sterile pipette tips in separate tubes containing 180 μl lysis buffer. After addition of 25 μl proteinase K to each sample, ticks and rodent samples were incubated overnight at 56 °C in a thermoshaker TS 100c (Biosan, Riga, Latvia). The next day DNA was extracted according to the manufacturer’s instructions. Quantity and quality of the extracted DNA samples were determined with a spectrophotometer Nanodrop 2000c (Thermo Scientific, Wilmington, USA). DNA lysates were stored at −20 °C prior to use.

### PCR amplification

The samples were screened for the presence of *A. phagocytophilum* DNA with a real-time polymerase chain reaction (PCR) targeting a 77-bp long fragment of the *msp2* (major surface protein) gene according to Courtney *et al.* [32]. The primers used were ApMSP2f (5′-ATGGAAGGTAGTGTTGGTTATGGTATT-3′) and ApMSP2r (5′-TTGGTCTTGAAGCGCTCGTA-3′) and the complementary probe was ApMSP2p TaqMan probe (5′-TGGTGCCAGGGTTGAGCTTGAGATTG-3′) labelled with 5′-HEX—TAMRA-3′. The PCR reaction was carried out in a reaction volume of 25 μl using a real-time PCR machine CFX96 Real-Time PCR System (Bio-Rad, Hercules, CA, USA) and a HotStarTaq PCR kit (Qiagen, Hilden, Germany). The PCR was set at the following parameters: denaturation at 95 °C for 5 min, 40 cycles of a denaturation period at 95 °C for 15 s and a 1 min annealing period at 60 °C. Negative and positive controls were included in each run.

Samples were considered positive with an exponential rise of the curve and a ct-value (threshold cycle) <37.5.

### Statistical analysis

Differences in *A. phagocytophilum* prevalence in questing ticks between sites, among years and between spring and autumn were computed by the Pearson *χ*^2^ goodness-of-fit or Fisher’s exact test in cases when the conditions for the Pearson *χ*^*2*^ goodness-of-fit test were not fulfilled. *P* < 0.05 was regarded as significant. The 95 % confidence intervals for prevalences in questing ticks were computed using a bootstrap technique. Logistic regression was used to estimate the effect of site, year, season and tick developmental stage on the probability of tick infection. Backward stepwise method was used to find the set of variables significantly affecting the probability of tick infection. Tests for the significance of the effects in the model were performed via the Wald statistic. Spearman rank correlation coefficient was calculated to estimate the relationship between prevalence of *A. phagocytophilum* and relative abundance of questing ticks and abundance of wildlife. Statistical analyses were performed with IBM SPSS Statistics, version 22 and Statistica software, version 12.

## Results

### Questing ticks

A total of 7984 questing ticks (nymphs and adults) were collected during the study: 4265 in Bratislava (4204 *I. ricinus* and 61 *H. concinna*) and 3719 in Fúgelka (3655 *I. ricinus*, 64 *H. concinna*). Among them, 5767 *I. ricinus* were collected in transects: 3224 (2676 nymphs, 548 adults) in Bratislava (transects B1, B2, B3) and 2543 (2119 nymphs, 424 adults) in Fúgelka (transects F1, F2, F3). The relative tick density varied among transects as well as among years and during the season. In Bratislava, the highest average density of nymphs was registered in transect B1, the lowest in transect B2, whereas the density of adults was lowest in transect B1 and highest in transect B2. In Fúgelka, the highest average density of nymphs was registered in transect F2 and the lowest in transect F1. However, the density of adults was highest in transect F2 and lowest in transect F3 (Table 1).

**Table 1:** Questing *I. ricinus* average relative density (number/100 m^2^) per transect in Bratislava and Fúgelka

Transect	Nymphs				Females				Males			
	2011	2012	2013	AVG	2011	2012	2013	AVG	2011	2012	2013	AVG
B1	159.5	69.7	119.4	116.9	1.5	1.0	4.1	2.6	3.8	3.0	5.1	4.2
B2	8.2	2.0	16.1	10.3	22.8	4.0	5.1	9.5	14.0	6.3	6.1	8.3
B3	57.2	24.5	63.5	51.4	5.8	7.3	5.4	6.0	9.8	7.8	2.7	5.9
F1	15.7	10.0	23.4	16.4	7.5	2.8	3.0	4.2	5.8	1.8	4.0	3.7
F2	112.0	74.2	45.4	74.8	9.3	6.6	4.6	6.6	14.3	8.8	4.2	8.7
F3	86.5	38.2	60.4	59.9	4.5	1.2	2.6	2.6	4.5	4.2	4.4	4.4

B1 − B3, transects in Bratislava; F1 − F3, transects in Fúgelka; AVG, average (2011–2013)

### Real-time PCR analysis of *A. phagocytophilum* in questing ticks

Out of the 4374 screened questing *I. ricinus* collected along six transects and their close vicinity, the presence of *A. phagocytophilum* DNA was detected in 224 ticks (5.1 %). Infected ticks were found in all locations. Overall prevalence of *A. phagocytophilum* was significantly higher in Bratislava (7.2 %; 95 % CI: 6.1–8.3 %) compared to Fúgelka (3.1 %; 95 % CI: 2.5–3.9 %) (*χ*^2^ = 37.451; *P* < 0.001). Significant differences in infection rates in questing ticks were also found among years at both sites (Table 2). In Bratislava, the lowest prevalence was observed in 2013 (4.3 %; 95 % CI: 2.9–5.7 %) and the highest in 2012 (9.3 %; 95 % CI: 6.2–12.7 %). Differences in prevalences among years were significant for all ticks (*χ*^2^ = 15.940; *P* < 0.001), females (*χ*^2^ = 8.444; *P* = 0.015) and nymphs (*χ*^2^ = 14.409; *P* = 0.001), but not for males (*χ*^2^ = 0.785; *P* = 0.675). At Fúgelka, the lowest *A. phagocytophilum* prevalence was found in 2011 (2.4 %; 95 % CI: 1.7–3.2 %) and the highest in 2013 (4.8 %; 95 % CI: 2.7–6.8 %). Significant differences among years were found in the total prevalence (*χ*^2^ = 7.227; *P* = 0.027) and prevalence for nymphs (*χ*^2^ = 15.777; *P* < 0.001), but not for prevalence in adults (females: Fisher’s exact test: *P* = 0.790; males: *χ*^2^ = 3.939; *P* = 0.140) (Table 2).

**Table 2:** Prevalence of *A. phagocytophilum* in *I. ricinus* per site in 2011–2013

Site		2011		2012		2013				Total
		% (pos/ex)	95 % CI	% (pos/ex)	95 % CI	% (pos/ex)	95 % CI	*χ* ^2^	*P*	% (pos/ex)
Bratislava	Nymphs	7.0 (48/686)	5.2–9.1	5.6 (11/195)	2.6–9.2	2.0 (9/455)	0.9–3.3	14.409	0.001	5.1 (68/1336)
	Females	15.6 (22/141)	9.7–22.1	18.0 (11/61)	8.2–27.9	6.4 (10/156)	2.6–10.3	8.444	0.015	12.0 (43/358)
	Males	10.7 (19/178)	6.4–15.5	11.8 (8/68)	4.4–19.1	8.5 (15/177)	4.5–12.4	0.785	0.675	9.9 (42/423)
	Total	8.9 (89/1005)	7.2–10.6	9.3 (30/324)	6.2–12.7	4.3 (34/788)	2.9–5.7	15.940	<0.001	7.2 (153/2117)
Fúgelka	Nymphs	0.8 (9/1067)	0.4–1.4	3.7 (11/295)	1.7–5.8	3.4 (9/263)	1.5–5.7	15.777	<0.001	1.8 (29/1625)
	Females	6.0 (9/150)	2.7–10.0	6.8 (4/59)	1.7–13.6	4.0 (3/75)	0.0–9.3		0.790^a^	5.6 (16/284)
	Males	9.1 (15/164)	4.9–14.0	2.4 (2/82)	0.0–6.1	8.8 (9/102)	3.9–14.7	3.939	0.140	7.5 (26/348)
	Total	2.4 (33/1381)	1.7–3.2	3.9 (17/436)	2.1–5.7	4.8 (21/440)	2.7–6.8	7.227	0.027	3.1 (71/2257)

(pos/ex), number of positive/number of examined; 95 % CI, confidence interval; *χ* ^2^, goodness-of-fit test; *P,* significance level

^a^Fisher’s exact test was used to compare prevalence in females among years because the condition of *χ* ^2^ goodness-of-fit test was not fulfilled

Overall *A. phagocytophilum* infection rate in questing adult ticks (9.0 %; 95 % CI: 7.6–10.6 %) was significantly higher than in nymphs (3.3 %; 95 % CI: 2.6–3.9 %) (*χ*^2^ = 64.231; *P* < 0.001). The same trend was observed in both habitats (Table 3).

**Table 3:** Overall prevalence of *A. phagocytophilum* in *I. ricinus* nymphs and adults per site

Site	Nymphs		Adults			
	% (pos/ex)	95 % CI	% (pos/ex)	95 % CI	*χ* ^2^	*P*
Bratislava	5.1 (68/1336)	3.9–6.2	10.9 (85/781)	8.9–12.9	24.675	<0.001
Fúgelka	1.8 (29/1625)	1.2–2.5	6.6 (42/632)	4.7–8.5	35.289	<0.001
Total	3.3 (97/2961)	2.6–3.9	9.0 (127/1413)	7.6–10.6	64.231	<0.001

(pos/ex), number of positive/number of examined; 95 % CI, confidence interval; *χ* ^2^, goodness-of-fit test; *P*, significance level

By comparing seasonal differences in overall prevalence of *A. phagocytophilum* in questing *I. ricinus* collected in the urban/suburban and natural habitat, significant differences were found between sites in the period from April to the end of June (spring-early summer) (*χ*^2^ = 44.470; *P* < 0.001), but not in the period from the end of July to October (late summer-autumn) (*χ*^2^ = 0.012; *P* = 0.913). Considering tick developmental stage and sex, in Bratislava, the highest prevalence of *A. phagocytophilum* was found in females and the lowest in nymphs collected in spring-early summer as well as in late summer-autumn (Table 4). In Fúgelka, the situation was different. In spring-early summer, the highest infection rate was found in males and the lowest in nymphs, whereas in late summer-autumn, the highest prevalence was found in females and the lowest in males. Comparing *A. phagocytophilum* infection rates in ticks collected in spring-early summer and late summer-autumn, significant differences were detected in nymphs at both sites (Bratislava: *χ*^2^ = 4.662, *P* = 0.031, Fúgelka: Fisher’s exact test, *P* = 0.037).

**Table 4:** Seasonal differences in prevalence of *A. phagocytophilum* in *I. ricinus*

Site		Spring-early summer		Late summer-autumn			
		% (pos/ex)	95 % CI	% (pos/ex)	95 % CI	*χ* ^2^	*P*
Bratislava	Nymphs	5.7 (62/1085)	4.3–7.1	2.4 (6/251)	0.8–4.4	4.662	0.031
	Females	12.7 (38/300)	8.7–17.0	8.6 (5/58)	1.7–15.5	0.753	0.386
	Males	10.4 (39/376)	7.2–13.6	6.4 (3/47)	0.0–14.9		0.603^a^
	Total	7.9 (139/1761)	6.7–9.1	3.9 (14/356)	2.0–5.9	6.928	0.008
Fúgelka	Nymphs	1.6 (23/1481)	0.9–2.2	4.2 (6/144)	1.4–7.6		0.037^a^
	Females	5.4 (14/257)	3.1–8.6	7.4 (2/27)	0.0–18.5		0.655^a^
	Males	8.0 (26/325)	5.2–11.1	0.0 (0/23)	-		0.239^a^
	Total	3.1 (63/2063)	2.3–3.8	4.1 (8/194)	1.5–7.2	0.666	0.414

Spring-early summer, April-end of June; Late summer-autumn, end of July-October; (pos/ex), number of positive/number of examined; 95 % CI, confidence interval; *χ* ^2^, goodness-of-fit test; *P*, significance level

^a^Fisher’s exact test was used to compare prevalence between spring and autumn because the condition of *χ* ^2^ goodness-of-fit test was not fulfilled

Simultaneous effect of site, year, season and tick developmental stage on the probability of infection was examined by logistic regression, resulting in significant effect of developmental stage (adults/nymphs; *P* < 0.001) and interaction of year and site (*P* < 0.001) on the prevalence of *A. phagocytophilum* in questing ticks (Table 5).

**Table 5:** Weight and significance of variables remaining in the best selected model for *A. phagocytophilum* prevalence in *I. ricinus*

Variable	B	S.E.	Wald	df	*P*	Exp(B)
Tick developmental stage (1 = Adults)	1.065	0.140	57.700	1	<0.001	2.901
Site*Year			52.132	2	<0.001	
Site1*Year 1	1.017	0.150	45.709	1	<0.001	2.764
Site1*Year 2	0.974	0.218	19.896	1	<0.001	2.648
Constant	–3.793	0.126	900.062	1	<0.001	0.023

Categorical variables codings: Site1, Bratislava; Year 1, 2011; Year 2, 2012; variables removed by backward method were year, site and season; B, parameter estimate; Wald, Wald statistic = test of significance of the regression coefficient; *P*, significance level

Except for differences in *A. phagoctytophilum* infection rates in ticks originating from different habitat types, overall prevalence differed significantly among transects within each habitat (Table 6). In Bratislava, the highest overall prevalence was detected in transect B2, in Fúgelka in transect F3 (Table 6).

**Table 6:** Overall prevalence of *A. phagocytophilum* in *I. ricinus* per transect in Bratislava and Fúgelka

	% (pos/ex)	95 % CI	% (pos/ex)	95 % CI	% (pos/ex)	95 % CI	*χ* ^2^	*P*
Bratislava/transect	B1		B2		B3			
	6.3 (53/836)	4.8–8.1	10.6 (57/539)	8.2–13.4	5.8 (43/742)	4.2–7.5	12.262	0.002
Fúgelka/transect	F1		F2		F3			
	3.1 (15/491)	1.6–4.7	1.9 (19/1018)	1.1–2.8	4.9 (37/748)	3.5–6.5	13.443	0.001

(pos/ex), number of positive/number of examined; 95 % CI, confidence interval; *χ* ^2^, goodness-of-fit test; *P*, significance level

Considering local differences in *A. phagocytophilum* infection rates, the results were separately evaluated for the urban/suburban habitat. *Ixodes ricinus* ticks were collected, in addition to the three transects sampled for three consecutive years, in two other transects in Bratislava (B4 and B5) in 2011. Comparison of infection rates between the five transects revealed significant differences for nymphs as well as adults. The highest overall prevalence of *A. phagocytophilum* was observed in transect B4 (35.4 %) (Table 7).

**Table 7:** Prevalence of *A. phagocytophilum* in *I. ricinus* per transect B1-B5 in Bratislava in 2011

	B1		B2		B3		B4		B5			
	% (pos/ex)	95 % CI	% (pos/ex)	95 % CI	% (pos/ex)	95 % CI	% (pos/ex)	95 % CI	% (pos/ex)	95 % CI	*χ* ^2^	*P*
Nymphs	4.7 (18/380)	2.6–7.1	26.0 (19/73)	16.2–36.7	4.7 (11/233)	2.2–8.0	23.2 (43/185)	17.3–29.7	0.0 (0/13)		75.198	<0.001
Females	14.8 (4/27)	3.3–29.6	15.6 (14/90)	8.5–23.4	16.7 (4/24)	3.4–35.0	45.5 (60/132)	37.1–53.8	14.7 (14/95)	8.4–22.1	40.087	<0.001
Males	13.1 (8/61)	5.9–21.7	9.2 (7/76)	3.0–15.8	9.8 (4/41)	2.2–20.9	41.0 (66/161)	33.6–48.4	9.2 (9/98)	4.1–15.3	57.731	<0.001
Total	6.4 (30/468)	4.2–8.8	16.7 (40/239)	12.2–21.5	6.4 (19/298)	3.5–9.5	35.4 (169/478)	30.8–39.5	11.2 (23/206)	6.8–15.5	183.125	<0.001

(pos/ex), number of positive/number of examined; 95 % CI, confidence interval; *χ* ^2^, goodness-of-fit test; *P*, significance level

Although the relative density of *I. ricinus* differed in individual transects within each habitat (see Table 1), infection rates of *A. phagocytophilum* did not correlate significantly with relative abundance of questing ticks (r_s_ = 0.201, *P* = 0.063). In the study area, positive but not significant correlation of *A. phagocytophilum* prevalence with abundance of roe deer was found (r_s_ = 0.576, *P* = 0.104). Negative non-significant correlations were found between prevalence and density of the other selected tick maintenance and potential reservoir hosts (red deer: r_s_ = -0.610, *P* = 0.081; fallow deer: r_s_ = -0.136, *P* = 0.728; mouflon: r_s_ = -0.492, *P* = 0.179), whereas significant negative correlation was found for the density of wild boar (r_s_ = -0.695, *P* = 0.038).

### Rodents and rodent-attached ticks

Altogether 300 rodents (138 females, 162 males) were caught in Bratislava, with dominance of the yellow-necked mouse (*Apodemus flavicollis*) (180 ind.: 83 females, 97 males), followed by bank vole (*Myodes glareolus*) (119 ind.: 54 females, 65 males) and one wood mouse (*Apodemus sylvaticus*: female). The majority of rodents were captured along transect B2 (153 ind.) and B3 (144 ind.). Only three rodent individuals were captured along transect B1. Altogether 306 rodents (147 females, 159 males) were caught at Fúgelka. The dominant species was *A. flavicollis* (176 ind.: 80 females, 96 males), followed by *M. glareolus* (108 ind.: 55 females, 53 males), common vole (*Microtus arvalis*) (19 ind.: 11 females, 8 males) and only one individual of harvest mouse (*Micromys minutus*: male), *A. sylvaticus* (female) and common pine vole (*Microtus subterraneus*: female). The majority of rodents were captured along transect F1 (166 ind.), whereas along transects F2 and F3, the number of captured rodents was 71 and 69 individuals, respectively. Comparing all three years, a substantial decline in rodent populations was observed in both sites in 2013. A total of 407 and 191 rodents were caught in 2012 and 2014, respectively, whereas only 8 individuals were caught in 2013.

In 2012, 764 ixodid ticks were collected from rodents: *Ixodes ricinus* (94.1 %) and *Haemaphysalis concinna* (5.9 %). In 2013, among the 207 rodent-attached ticks *I. ricinus* prevailed (99.5 %) and *H. concinna* represented only 0.5 %. In 2014, altogether 1031 ticks were collected from rodents: *I. ricinus* (97.2 %), *H. concinna* (2.3 %), *Ixodes trianguliceps* (0.4 %; found only in Bratislava Forest Park, transect B3) and *Dermacentor reticulatus* (0.1 %; found only in Fúgelka, transect F1).

### Real-time PCR analysis of *A. phagocytophilum* in rodents and rodent-attached ticks

Spleen and skin samples from rodents as well as rodent-attached ticks were tested for the presence of *A. phagocytophilum*. The bacterium was detected in skin of three rodents (0.5 %; 3/606) and only in one spleen sample (0.2 %; 1/606). All positive samples came from bank voles trapped in Bratislava Forest Park (transect B3). In one of the above mentioned specimens, *A. phagocytophilum* was detected in both skin and spleen simultaneously.

In total, 0.9 % (5/553) and 0.2 % (1/445) of tested engorged ticks were positive for *A. phagocytophilum* from rodents captured in Bratislava and Fúgelka, respectively (Table 8). Analysing the tested rodent-attached ticks, only *I. ricinus* carried the bacterium, *H. concinna, I. trianguliceps* and *D. reticulatus* were negative. One positive tick larva was removed from the bank vole specimen with *A. phagocytophilum*-positive skin and spleen (trapped in Bratislava, B3). All four positive nymphs were removed from the same yellow-necked mouse specimen trapped in Bratislava (transect B2) and one positive larva was removed from a yellow-necked mouse trapped in Fúgelka. However, skin and spleen of both yellow-necked mice were negative.

**Table 8:** Prevalence of *A. phagocytophilum* in rodent-attached ticks captured per site in 2012–2014

Site	Year	*I. ricinus* pos/ex			*H. concinna* pos/ex		*I. trianguliceps* pos/ex		*D. reticulatus* pos/ex	Total % (pos/ex)
		L	N	A	L	A	L	N	L	
Bratislava	2012	1/213			0/17					0.4 (1/230)
	2013	0/30	4/21			0/1				7.7 (4/52)
	2014	0/262			0/5		0/2	0/2		0.0 (0/271)
	Total	1/505	4/21		0/22	0/1	0/2	0/2		0.9 (5/553)
Fúgelka	2012	1/169	0/1	0/1	0/16	0/3				0.5 (1/190)
	2013	0/5								0.0 (0/5)
	2014	0/226	0/5		0/18				0/1	0.0 (0/250)
	Total	1/400	0/6	0/1	0/34	0/3			0/1	0.2 (1/445)
Total		2/905	4/27	0/1	0/56	0/4	0/2	0/2	0/1	0.6 (6/998)

pos/ex, number of positive/number of examined; L, larva; N, nymph; A, adult

## Discussion

### *Anaplasma phagocytophilum* in questing ticks

In the present study the prevalence of *A. phagocytophilum* was examined in questing ticks and rodents in two ecologically contrasting habitat types in south-western Slovakia. Infected questing *I. ricinus* ticks were found in all tick collection locations within each habitat. The total prevalence was 5.1 %, which is higher than recently reported for other sites in south-western Slovakia (1.0–4.1 %) [33]. Infection rates between 0.7–13.3 % were determined in *I. ricinus* from different regions of Slovakia [13, 15, 27, 33–40]. In Europe, *A. phagocytophilum* prevalence in questing *I. ricinus* was found to vary from 0 % to 34 % [3, 16]. Recent studies revealed overall prevalences that varied from 0.4 % in Hungary [41], 0.6 % in Slovenia [42], 0.8 % in the Netherlands [43], 1.5 % in Switzerland [44], 1.9 % in Luxembourg [45], 3.6 % in Ukraine [46], 5.3 % in Germany [18] and up to 8.5 % in Poland [47, 48]. Generally, prevalence data based on molecular detection of *A. phagocytophilum* in questing ticks have been found to vary considerably not only between countries, but also among habitat types and sites within each country.

In this study, *I. ricinus* relative abundance varied among locations in each habitat, but overall abundance was higher in the urban/suburban habitat. The overall *A. phagocytophilum* prevalence was also significantly higher in the urban/suburban habitat (7.2 %) compared to the natural habitat (3.1 %). Our results are in contrast, e.g., to findings from Poland, where tick abundance was higher in natural habitats [48], but are in agreement with findings from Germany or eastern Slovakia, where abundant tick populations are present in urban parks and suburban forests [18, 40]. Our findings are also in accordance with observations from Central European urban, suburban and natural sites and support the theory that prevalence of *A. phagocytophilum* depends on the habitat type and the diversity and population density of potential reservoir hosts [4, 19, 49]. Relatively high percentage of urban/suburban *I. ricinus* populations were found to be infected by *A. phagocytophilum* throughout Europe, e.g., 2.9 % to 9.5 % in urban/suburban parks in Germany [18, 19, 23, 49], 5.8 % to 9.4 % in the Czech Republic [20, 33], 8.8 % in Hungary [50], up to 14.5 % in Poland [47, 48], 4.4 % to 7.5 % in urban parks in Bratislava and Malacky (south-western Slovakia) [38, 51], but only 1.0 % in Austria [52] and 0.8 % in Luxembourg [45]. In contrast, prevalence of *A. phagocytophilum* was generally lower in natural woodland habitats, e.g., 1.0 % to 5.8 % in Germany [12, 19, 53–55], 1.9 % in the Czech Republic [20], 0 % to 2.2 % in western Slovakia [38] and 0.4 % in Hungary [41], whereas it was higher (1.3 %) than in urban sites in Luxembourg [45].

In addition to the variation in *A. phagocytophilum* prevalence between the two habitat types, local differences among sampling locations within each habitat were revealed in this study. Our findings corroborate local variations, e.g. in Bavarian cities [19, 49], Hannover [56] and Leipzig [23], or in Budapest [50], and are probably connected with the specific structure of the biotope in each individual location. The high number of positive ticks in transect B4 in Bratislava (35.4 % overall infection rate with more than 40.0 % of adult ticks infected in 2011) deserves particular attention and could be due to the presence of roe deer in the enclosed area of the SAS campus.

Generally, wild ruminants, mainly roe deer, have shown high infection rates with *A. phagocytophilum* and have been suggested as reservoir hosts [12, 57–62]. Based on the high relative abundance of *I. ricinus* in the SAS campus (transect B1, see Table 1), we assume that roe deer support an abundant tick population and may contribute to the maintenance of *A. phagocytophilum* at this location. In addition, our results show that even within a small enclosed area significant local variations in distribution of infected ticks can occur: in transect B4 a prevalence of 35.4 % was determined in 2011, whereas in transect B1 (the distance between B1 and B4 is about 350 m) only 6.4 % of ticks were infected. Even lower prevalence (5.8 %) was found in another location in the SAS campus during 2011–2012 [63]. Nevertheless, the high proportion of infected ticks is not direct evidence for maintenance of *A. phagocytophilum* by roe deer in the SAS campus. Examination of blood or xenodiagnostic ticks has not been possible due to legislative and ethical constraints. However, our results support previous findings on city parks being focal points for *A. phagocytophilum* [19]. Furthermore, our results are in agreement with higher infection rates in questing ticks found in sites with high densities of roe deer [64]. The non-significant relationships between prevalence rates and abundance of other cervids in our research area seem to support findings in a study [65], where no clear association between density of red deer, another potential reservoir host, and prevalence of *A. phagocytophilum* could be confirmed. Wild boars are abundant game animals that can feed on all three life stages of *I. ricinus* and might be involved in the enzootic cycle of *A. phagocytophilum* [66]. Although wild boar is susceptible to *A. phagocytophilum*, it can control the infection and thus its role as reservoir for the bacterium and source of infection for ticks is probably not as significant as suggested by some studies [67]. The negative correlation between wild boar abundance and prevalence of infected ticks in our study area seems to support the latter assumption. However, further studies involving molecular screenings of wild boar samples and attached ticks are required.

Generally, reduced vertebrate biodiversity was found to affect transmission of infectious diseases of humans and animals, frequently leading to increased disease transmission in urban areas. The urban (closed) habitat comprises fewer host species which usually occur in higher densities and in case they are competent reservoirs, they can support pathogen transmission [4, 68]. We assume that the higher prevalence of *A. phagocytophilum-*infected ticks in Bratislava Forest Park, compared to the natural habitat in Fúgelka, could be partly affected by higher population densities of roe deer and hedgehogs which are known to carry the pathogen in urban parks [4]. The presence of a large number of dog owners who use Bratislava Forest Park for their daily walks with dogs may be important as well. Seroprevalences of up to 50.1 % detected in dogs from different European countries suggest that they could play a role in the epidemiology of *A. phagocytophilum* [69]*.* In contrast, due to higher diversity of vertebrate species in the woodland area at Fúgelka ticks can feed on a broader range of host species, among which a lower proportion may support circulation of *A. phagocytophilum.*

Along with spatial variations, pathogen prevalence in questing ticks was found to vary in time, i.e. between developmental stages of ticks, during the season and among years at the same study sites [18, 41, 43, 45, 49, 56, 70]. In the present study infection rates were higher in adult ticks (5.6–12.0 %) than in nymphs (1.8–5.1 %). The results confirm findings of previous studies [16, 18, 19, 48, 49, 71] and could be explained by the feeding strategy of three-host ticks enabling them to acquire infected blood meals in larval as well as nymphal stage and transmit pathogens transstadially. Our study also revealed significant seasonal and year-to-year variations in individual tick collection locations, mainly for prevalence of infected nymphs. However, temporal variations in pathogen prevalences are difficult to explain, especially for *I. ricinus,* having a complex life history lasting for several years. Variations of this kind may depend on global factors affecting all study sites, such as weather conditions during the years and seasons. In individual locations temporal variations can be determined by microclimatic conditions, phenology of the tick and the availability of feeding and reservoir hosts [12, 18, 43, 45, 56].

In addition to *I. ricinus,* the main vector of *A. phagocytophilum* in Europe [3], we detected the bacterium in one questing *H. concinna* male. Single detections of *A. phagocytophilum* in questing *H. concinna* have already been reported from Slovakia [34] and Serbia [72], but not from Hungary [73, 74]. Such sporadic data, however, do now allow conclusions to be drawn about the vector status of this tick species.

### *Anaplasma phagocytophilum* in rodents and rodent-attached ticks

Apart from wild ruminants *A. phagocytophilum* has been detected in rodents as the major feeding hosts of subadult stages of *I. ricinus* [3, 4]. Although several studies have reported the role of rodents in transmission of *A. phagocytophilum* in Europe*,* some consider rodents as reservoirs of the pathogen [7, 9–11, 75], in this study we found that only 0.5 % of rodents were infected. The positive samples originated from bank voles trapped in Bratislava Forest Park. In other regions of Slovakia, the prevalence of *A. phagocytophilum* in rodents varied from 0 to 9.3 % [11, 13, 15, 36, 76]. Prevalence rates between 0 and 6.6 % have recently been reported for rodents from Hungary, Germany and Italy [14, 23, 25, 73, 74]. In contrast, the prevalence of infected rodents, especially bank voles, was higher in Switzerland and Germany (over 19.0 and 13.0 %, respectively) [7, 75].

In our study sites, the low overall prevalence of *A. phagocytophilum* in rodents is in contrast to the relatively high prevalence in questing ticks and could be explained by the specific host tropism of *A. phagocytophilum* strains circulating in the study area. Constant detection of the bacterium in questing ticks throughout the study in contrast to low rodent density in the SAS campus (J. Krištofík, personal communication) and a sharp decline in rodent abundance in all locations in 2013 seem to support this assumption.

Despite the fact that questing *I. ricinus* larvae are considered *A. phagocytophilum-*free, because no transovarial transmission occurs [17], we have detected two positive rodent-attached larvae (0.1 %). One larva was collected from an infected bank vole with positive skin and spleen which was captured in transect B3. The other one was gained from an uninfected yellow-necked mouse captured in the natural site. Moreover, four *I. ricinus* nymphs from an uninfected yellow-necked mouse captured in transect B2 were pathogen-positive. According to recent studies, the prevalence of *A. phagocytophilum* in engorged *I. ricinus* ticks varies from 0 to 6.1 % in Europe [14, 25, 73, 74, 77], however,in field-captured tick-infested rodents no transmission of the bacterium to xenodiagnostic *I. ricinus* larvae was confirmed [77, 78]. Rodents were found to be infected with a distinct non-pathogenic strain of *A. phagocytophilum* in areas where the endophilic *I. trianguliceps* ticks are present [10, 13, 79]. We found only a few non-infected *I. trianguliceps* specimens infesting rodents, suggesting that small populations of the species are present in the Small Carpathian Mountains and may be involved in the circulation of a rodent-specific *A. phagocytophilum* strain in the area. However, due to low level of infection in the positive bank vole and attached tick larvae (ct-values >30 in real-time PCR detections) we were not able to confirm this assumption by analysing the *A. phagocytophilum* DNA for strain specificity. We assume that the few positive rodent-attached *I. ricinus* nymphs acquired infection by feeding on infected hosts in the larval stage and the single larva feeding on an uninfected yellow-necked mouse gained infection via feeding on an infected host and after interruption continued feeding on the rodent.

## Conclusions

During a three-year study in south-western Slovakia, constant presence of *A. phagocytophilum* in questing *I. ricinus* was confirmed with infection rates depending on habitat type, location, year, season and availability of tick hosts and pathogen reservoirs. The study confirmed that urban *I. ricinus* populations are infected with the bacterium at a significantly higher rate than in a woodland habitat. Moreover, the results indicate that urban/suburban sites and parks can be focal points for *A. phagocytophilum* circulation in south-western Slovakia. In contrast to the relatively high prevalence of *A. phagocytophilum* in questing ticks, infection rates in both rodents and rodent-attached ticks were low (<1 %), suggesting that rodents are not the main reservoirs of the bacterium in the investigated area. To assess the risk of infection of humans and domestic animals with pathogenic strains of *A. phagocytophilum,* further follow-up studies are required to analyse bacterial strains circulating in south-western Slovakia and their associations with reservoir hosts.
